# Relative Low Flow Extra Corporeal Co_2_-Removal in Ards Patients: A Pilot Study

**DOI:** 10.1186/2197-425X-3-S1-A513

**Published:** 2015-10-01

**Authors:** H Peperstraete, S Eloot, P Depuydt, C Roosens, F De Somer, E Hoste

**Affiliations:** Ghent University Hospital, Intensive Care, Gent, Belgium; Ghent University Hospital, Nephrology, Ghent, Belgium; Ghent University Hospital, Cardiac Surgery, Ghent, Belgium

## Introduction

Mechanical ventilation (MV) of patients with Acute Respiratory Distress Syndrome (ARDS) should be performed with a lung protective strategy, since this is associated with better clinical outcomes. Lung protective MV contains the lowering of the plateau pressure (P_PLAT_) and the tidal volume (V_T_). Physician's choice for lung protective MV can be hindered by the consequence of decreased CO_2_ clearance, i.e. respiratory acidosis.

Veno-venous extracorporeal CO_2_-removal (ECCO_2_-R) is a recent therapy allowing extracorporeal CO_2_ clearance and normalisation of pH.

## Objectives

The aim of this pilot study was to evaluate whether ECCO_2_-R using relative low blood flow was able to treat respiratory acidosis in ARDS patients treated with lung protective MV, so that further reduction of P_PLAT_ and V_T_ was feasible.

## Methods

This is a single centre trial in which patients who met the Berlin definition of ARDS with a PaO_2_/FiO_2_ < 150mmHg and who had respiratory acidosis were included. the first 2 hours of therapy blood flow was 300ml/min, after which it was increased to 400ml/min. During the ECCO_2_-R we aimed at lowering P_PLAT_ and V_T_. For every patient we used the Abylcap^®^ device (Bellco, Italy) with either the Lynda^®^ machine (8 patients) or the Amplya**™** (1 patient). Every patient was heparinized to prevent clotting of the circuit and oxygenator. During the complete study period, ventilator settings and results of blood gases were recorded. Data are reported as median [interquartile range] or n (%).

## Results

We included 9 patients, 4 female, with a median age of 50 y [22.8, 66.5]. All patients showed a decrease of pCO_2_ after 2 hours of treatment with median reduction of 28.2% [11.6, 31.0; p = 0.008]; five patients (56%) had a decrease in pCO_2_ of more than 20%. the median reduction in P_PLAT_ after 5 days (D5) of treatment was 8.5cmH_2_O (5.3, 12.5; p = 0.012). Median reduction in V_T_ at D5 was 1.52ml/kg predicted body weight (0.65, 1.85; p = 0.017). in all patients pH could be corrected to normal range values with an increase of median pH from 7.17 (7.11, 7.21) at inclusion to 7.42 (7.40 ,7.44) (p = 0.012) at D5. ECCO_2_-R was hemodynamically well tolerated. Three patients needed a blood transfusion because of bleeding. Two patients needed a circuit renewal earlier than scheduled because of clotting of the circuit or oxygenator, both patients were treated with the Lynda^®^ machine.

## Conclusions

In patients with moderate ARDS, veno-venous ECCO_2_-R using relative low blood flow is a promising extracorporeal technique allowing removal of CO_2_, thus allowing MV with lower P_PLAT_ and V_T_. An explanation for the inter-patient variation in efficiency of CO_2_ removal could not be found in our patient cohort.

## Grant Acknowledgment

Financial support by Bellco.

